# Oxygen metabolism abnormalities and high-altitude cerebral edema

**DOI:** 10.3389/fimmu.2025.1555910

**Published:** 2025-03-19

**Authors:** Zhi Li, Jianping Zhang, Xiaoxia Zhang, Qiaoying Jin, Xingxing Zheng, Li Mo, Zejiao Da

**Affiliations:** ^1^ The Second Hospital & Clinical Medical School, Lanzhou University, Lanzhou, Gansu, China; ^2^ State Key Laboratory of Ophthalmology, Zhongshan Ophthalmic Center, Sun Yat-Sen University, Guangzhou, Guangdong, China; ^3^ Department of Ophthalmology, Minxian People’s Hospital, Minxian, Gansu, China

**Keywords:** oxygen metabolism, hypobaric hypoxia, oxidative stress, mitochondrial dysfunction, high-altitude cerebral edema

## Abstract

Hypobaric hypoxia is widely recognized as a prominent risk factor for high-altitude cerebral edema (HACE), which contributes to the exacerbation of multiple pathological mechanisms, including oxidative stress, mitochondrial dysfunction, disruption of blood−;brain barrier integrity, neuroinflammation, and neuronal apoptosis. Among these mechanisms, abnormalities in oxygen metabolism, including hypoxia, oxidative stress, and mitochondrial dysfunction, play pivotal roles in the pathophysiology of HACE. In this review, our objective is to enhance our comprehension of the underlying molecular mechanisms implicated in HACE by investigating the potential involvement of oxygen metabolism. Addressing aberrations in oxygen metabolism holds promise for providing innovative therapeutic strategies for managing HACE.

## 1 Introduction

High altitude, defined as an elevation of 2500 m above sea level, encompasses approximately one-fifth of the Earth’s surface. According to a report in The Lancet, more than 140 million individuals resided at high altitudes above 2500 m as early as 2001 (1). Inadequately acclimatized individuals exposed to high altitudes may experience various high-altitude illnesses, including acute mountain sickness, high-altitude cerebral edema (HACE), and high-altitude pulmonary edema (2, 3). Despite its low incidence rate, HACE is a rapidly progressive and potentially fatal condition. Without prompt and effective treatment, patients can deteriorate rapidly within hours, leading to coma or even death (4). The pathophysiology of HACE remains incompletely understood; however, accumulating evidence suggests that it involves multiple factors. These include oxidative stress (5), cerebral blood flow disorders, disruption of blood−;brain barrier (BBB) integrity (6), inflammation (7, 8), REDOX homeostasis imbalance (9), mitochondrial dysfunction and neuronal apoptosis (10).

Hypobaric hypoxia (HH), the primary characteristic of a high-altitude environment, leads to reduced oxygen delivery to tissues (11). Owing to the heavy reliance of the brain on a consistent oxygen supply, it becomes vulnerable to fluctuations in oxygen levels. Consequently, abnormalities in oxygen metabolism play a pivotal role in the pathophysiology of HACE. The intricate interplay of molecular events governing oxygen metabolism may significantly contribute to the pathogenesis and progression of HACE. Therefore, this review places particular emphasis on elucidating the molecular mechanisms underlying recent discoveries related to abnormalities in oxygen metabolism associated with HACE, encompassing hypoxia, oxidative stress, and mitochondria. This comprehensive analysis has the potential to enhance our understanding of the correlation between HACE and oxygen metabolism while facilitating therapeutic approaches targeting these metabolic abnormalities for effective management.

## 2 Oxygen metabolism and HACE

The process of oxygen metabolism, which is vital for maintaining normal physiological functions in cells and tissues, involves a series of intricate mechanisms involved in the transfer, transportation, and utilization of oxygen. This metabolic pathway converts oxygen into adenosine triphosphate (ATP) to provide energy, while a small fraction is transformed into reactive oxygen species (ROS) (11). However, the aberrant accumulation of ROS can disrupt organismal functionality. The mitochondrion plays a crucial role in cellular oxygen consumption, exerting an influence on redox potential and governing the levels of ROS.

**Figure 1:**
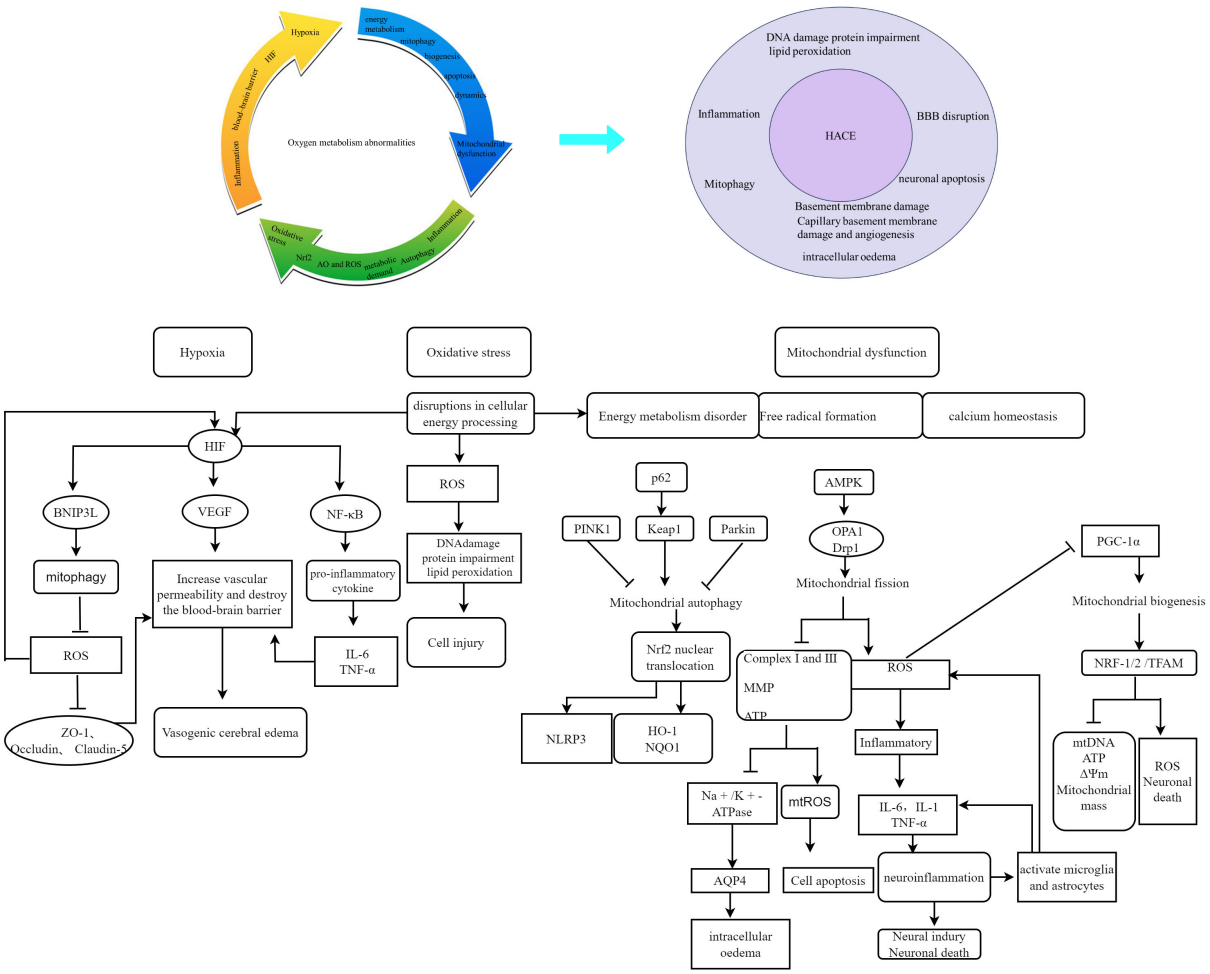
Schematic representation of the contribution of oxygen metabolism abnormalities to HACE pathology. Oxygen metabolism abnormalities constitute a significant risk factor in the pathogenesis and progression of HACE. Recent studies have highlighted hypoxia, oxidative stress, and mitochondrial dysfunction as pivotal components in both HACE and oxygen metabolism research. The interplay between oxygen metabolism disturbances and HACE pathology significantly contributes to disease deterioration.

Aberrations in oxygen metabolism predominantly include hypoxia, excessive ROS accumulation, and mitochondrial dysfunction. These factors can disturb physiological processes or initiate disease development (11). Upon exposure to a high-altitude hypoxic environment, diminished ambient oxygen availability induces an increase in ROS production within brain tissue. This phenomenon is positively associated with altitude. ROS exhibit strong reactivity and have the ability to oxidize lipids, proteins, and DNA, leading to alterations in cellular structure and function (12). The ROS target polyunsaturated fatty acids within biofilms, initiating a cascade of lipid peroxidation reactions. This leads to biofilm dysfunction and damage to membrane-bound enzymes (13). The excessive generation of ROS further exacerbates the oxidative stress response and disrupts the redox balance, ultimately resulting in cerebral injury (14). The presence of ROS in the brain can contribute to neuronal loss and cerebral edema (15). Elevated levels of ROS can also result in mitochondrial impairment, DNA alterations, augmented cytokine production, and even cellular apoptosis. (16, 17). Conversely, hypoxia-induced mitochondrial autophagy, which is regulated by the expression of BCL2-interacting protein 3-like (BNIP3L) via hypoxia-inducible factor (HIF), functions as an adaptive metabolic response to prevent excessive levels of ROS and maintain cellular survival (18). Therefore, abnormal oxygen metabolism is a significant factor in the development of HACE. **Figure 1** illustrates the contribution of oxygen metabolism abnormalities to HACE pathology.

## 3 Hypoxia and HACE

### 3.1 Hypoxia-inducible factor

**Figure 2:**
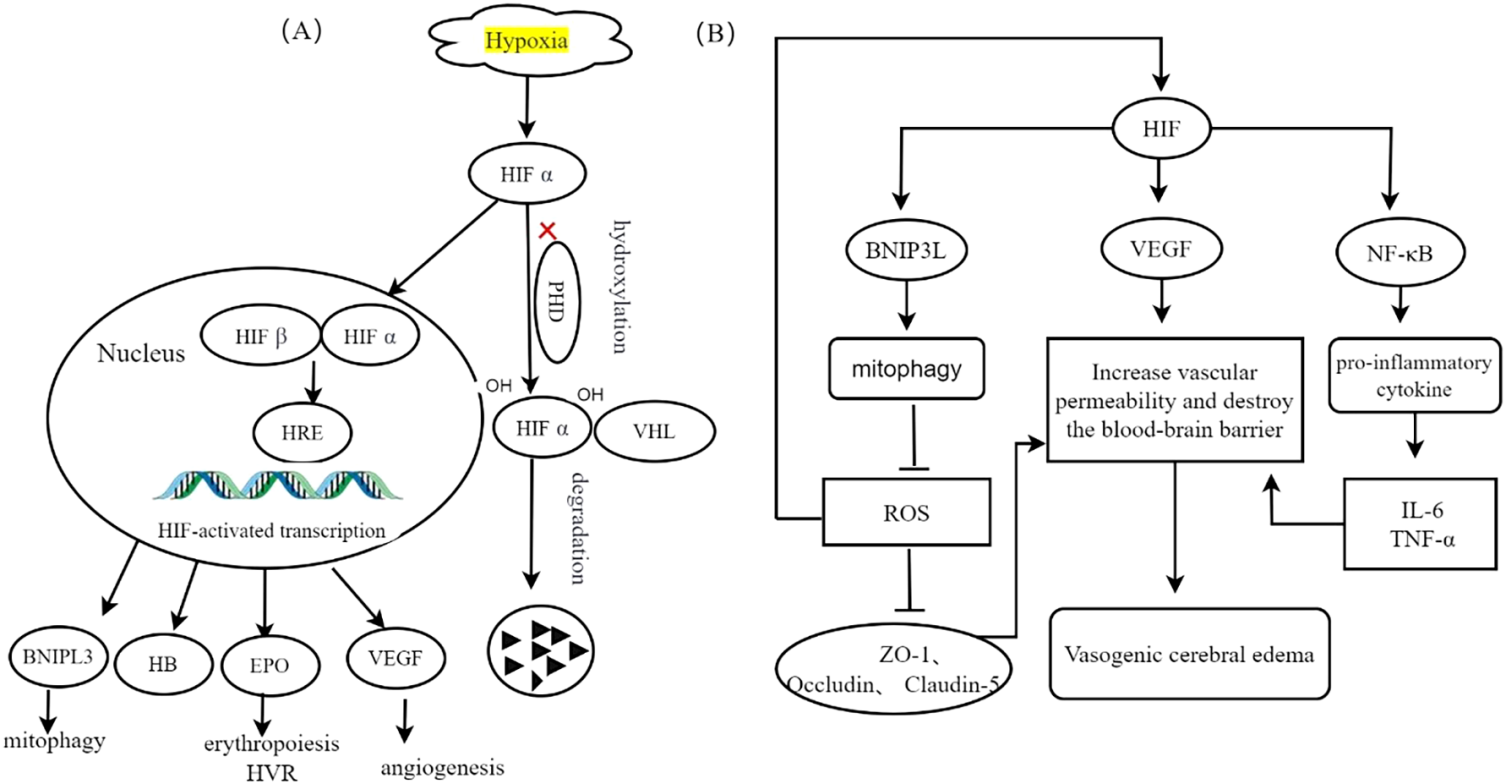
Transcriptional regulation of HIF induced by hypoxia, adapted from (3). **(A)** Hypoxia inhibits the proteasomal degradation of HIF-α and promotes its translocation into the nucleus. Following interaction with HIF-β, this complex synergistically activates the transcription of target genes, thereby enhancing cellular adaptation to hypoxic conditions. **(B)** The specific role of HIF-1α in different cell types is discussed. In mitochondria, elevated levels of HIF enhance the upregulation of BNIP3L, resulting in increased mitophagy and decreased ROS production. Elevated ROS levels compromise the integrity of cellular tight junction proteins, including ZO-1, Occludin, and Claudin-5, which in turn increases vascular permeability and contributes to vasogenic cerebral edema. In endothelial cells, the accumulation of HIF-1 results in the upregulation of VEGF, consequently increasing vascular permeability, compromising the integrity of the blood-brain barrier, and inducing vasogenic cerebral edemaIn microglia, HIF activation of the NF-κB signaling pathway facilitates the release of proinflammatory cytokines, such as IL-6, IL-1β, and TNF-α, increases vascular permeability, and contributes to cerebral edema.

The limited availability of oxygen at high altitudes triggers modifications in gene expression through the HIF pathway, which plays a crucial role in the cellular response to hypoxia. HIFs are heterodimers composed of one of three hypoxia-inducible α subunits (HIF-1α, HIF-2α, or HIF-3α) and a constitutively expressed β subunit (HIF-β). Under normal oxygen conditions, HIF-α undergoes hydroxylation facilitated by interactions with prolyl hydroxylase domain (PHD) proteins, which provide a binding site for the von Hippel−;Lindau (VHL) protein and lead to degradation of HIF-α. However, under low-oxygen conditions, hydroxylation is inhibited, resulting in stabilized dimerization of HIF-α with HIF-β and subsequent binding to numerous target genes to initiate the transcriptional activation of key factors, such as erythropoietin (EPO), hemoglobin (HB), and vascular endothelial growth factor (VEGF) (19). **Figure 2** illustrates the regulation of HIF in response to hypoxic conditions.

#### 3.1.1 HIF-regulated erythropoietin

The EPAS1 gene, which encodes HIF-2α, has been identified as the predominant isoform involved in high-altitude adaptation on the basis of recent genome-wide studies (20). EPAS1 has a tissue- and cell-specific distribution and is expressed primarily in organs such as the brain (21). Hypoxic stabilization of HIF-2α at high altitudes leads to the formation of the HIF-2 complex, which activates EPO expression. EPO is a glycoprotein hormone primarily produced by the kidney that stimulates erythropoiesis in the bone marrow, thereby increasing the oxygen-carrying capacity of the blood. In the brain, EPO modulates both central and peripheral neural respiratory centers, leading to an augmented hypoxic ventilatory response (HVR) and improved tissue oxygenation.

#### 3.1.2 HIFs regulate vascular permeability factors

VEGF is an endothelial-specific mitogen that plays a crucial role in promoting angiogenesis through various mechanisms, including migration, proliferation, differentiation, and extracellular matrix proteolysis. It acts as a downstream effector of HIF-1. After 24 hours of acute exposure to HH, the expression of VEGF in the brain increased. Additionally, prolonged exposure to elevated levels of VEGF can lead to disruption of endothelial barrier integrity and increased vascular permeability, resulting in cerebral damage and the development of high-altitude cerebral edema (22). Furthermore, the activity of VEGF closely correlates with that of HIF-1α under hypoxic conditions, which further exacerbates inflammatory responses (23). Hypoxia-induced upregulation of HIF-1α serves as a pivotal regulatory mechanism that enhances subsequent VEGF expression, thereby promoting vasogenic cerebral edema.

#### 3.1.3 HIF regulated mitophagy

The BNIP3L protein, known as BCL2-interacting protein 3-like, functions as a specific receptor responsible for recognizing mitophagy while being localized on the outer membrane of mitochondria. Under hypoxic conditions, BNIP3L plays an essential role in promoting autophagy induced by low oxygen levels and ensuring cell survival (24). Hypoxia significantly increases BNIP3 expression through direct transcriptional targeting by HIF-1α. Elevated levels of HIF intensify the upregulation of BNIP3L, consequently leading to enhanced mitophagy induction and reduced production of ROS (25).

HIF-a, hypoxia inducible factor α-subunit; HIF-β, hypoxia inducible factor β-subunit; PHD, prolyl hydroxylase; VHL, Van Hippel–Lindau proteins; HRE, hypoxia response element; VEGF, vascular endothelial growth factor; EPO, erythropoietin; HVR, hypoxic ventilatory response; HB, hemoglobin;BNIF3, BCL2 interacting protein

### 3.2 Hypoxia and blood–brain barrier dysfunction

The BBB functions as a crucial protective shield for the central nervous system, ensuring the stability of the brain microenvironment (26). The disruption of the BBB induced by hypoxia is considered a primary contributing factor to the development of HACE (27, 28). This disruption results in elevated BBB permeability, and its severity directly correlates with the degree of cerebral edema observed on T2-weighted MR images in both HACE patients and animal models (29, 30). The occurrence of white matter edema in HACE signifies impaired function or disruption of the blood−;brain barrier, potentially resulting from membrane destabilization and inflammation induced by ROS or localized activation of HIF and VEGF (6).

HH stimulation disrupts blood−;brain barrier integrity by compromising the levels of the cellular tight junction proteins ZO-1, Occludin and Claudin-5 and increasing vascular permeability, resulting in vasogenic cerebral edema (6). Additionally, this stimulation induces the production of VEGF via HIF-1α, resulting in vascular injury (6, 7). Consequently, an inflammatory response is triggered, ultimately culminating in cytotoxic brain edema. Inflammation plays a pivotal role in the pathogenesis of cerebral edema, as inflammatory mediators (ET-1, TNF-α, and IL-1β) act on angiotropic endothelial cells within brain tissue to increase BBB permeability (31). The exposure of animals to HH combined with lipopolysaccharide (LPS) treatment likely synergistically affects BBB integrity and function, which is characterized by a compromised oxygen supply and exacerbated inflammatory response (32). Reinforcing the integrity of the BBB has thus emerged as a potent strategy for pharmacological intervention in cerebral edema.

**Figure 3:**
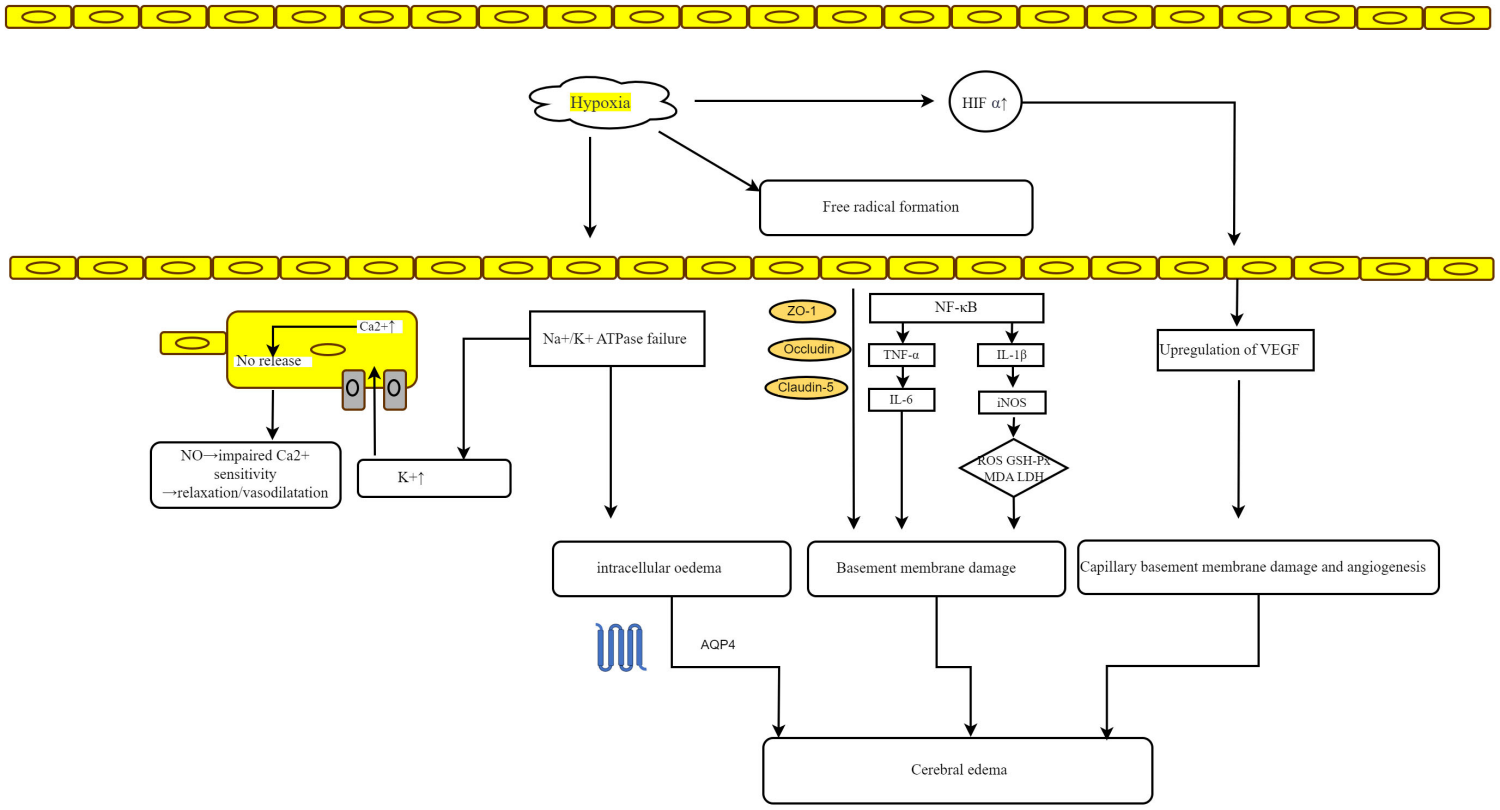
Mechanisms hypothesized to underlie HACE. Hypoxia-induced failure of Na+/K+ ATPase results in local hyperkalemia, which may trigger calcium-mediated nitric oxide release, leading to vasodilation in vascular smooth muscle. Additionally, ATPase failure under hypoxic conditions can cause intracellular edema. The formation of free radicals disrupts cellular tight junction proteins (ZO-1, Occludin, and Claudin-5) and triggers the release of inflammatory mediators, thereby increasing vascular permeability and damaging vessel basement membranes, ultimately resulting in vasogenic edema. The accumulation of HIF-1α and subsequent upregulation of VEGF further contribute to basement membrane damage and edema. Moreover, alterations in endothelial cells, including increased pinocytotic vesicles and disassembly of interendothelial tight junction proteins, as well as swelling of astrocyte endfeet, may also increase capillary permeability.

Microglia, which are a type of tissue-resident macrophage in the brain, serve dual functions in preserving the integrity of the BBB. Resting microglia support BBB maintenance, whereas activated microglia compromise BBB integrity by migrating toward blood vessels and phagocytosing the tight junctions of vascular endothelial cells, thereby increasing vascular permeability and inducing vasogenic cerebral edema (33, 34). Moreover, the release of proinflammatory factors from activated microglia and subsequent astrocyte swelling contribute to cytotoxic cerebral edema in HACE (33, 35). Consequently, inhibiting microglial activation may represent a promising therapeutic strategy for HACE (33). **Figure 3** elucidates the proposed mechanisms that are hypothesized to underlie HACE.

### 3.3 Hypoxia and inflammation

Hypoxia can induce a cerebral inflammatory response and facilitate the initiation and progression of cerebral edema in mice exposed to high-altitude conditions. A growing body of evidence emphasizes the pivotal role of inflammation in the progression of HACE (7, 28). A study by Song et al. demonstrated that exposure of volunteers to an altitude of 3860 m resulted in a significant increase in the plasma levels of TNF-α, IL-1β, and IL-6 (7). Proinflammatory cytokines, namely, IL-6 and IL-1β, were induced from the first day of high-altitude exposure onward according to Chauhan et al. (36).

HH triggers the activation of the NF-κB signaling pathway and facilitates the release of proinflammatory cytokines, such as IL-6, IL-1β, and TNF-α, leading to detrimental inflammatory responses within the central nervous system (35, 37, 38). The NF-κB signaling pathway plays a crucial regulatory role in the development of cerebral edema induced by high-altitude exposure (39)]. First, the expression of aquaporin 4 (AQP4) is directly controlled by the NF-κB signaling pathway. Wang suggested that puerarin exerts an anti-inflammatory effect by inhibiting the activation of the NF-κB pathway, thereby reducing AQP levels in the cerebrum (40). Second, the NF-κB signaling pathway significantly regulates the inflammatory response mediated through IL-1β, IL-6, and TNF-α generation during high-altitude exposure (41). Activation of the NF-κB signaling pathway was observed in the cerebral cortex of mice exposed to simulated high-altitude conditions as well as in astrocytes or microglia exposed to hypoxia. This activation was characterized by increased P65 phosphorylation and subsequent nuclear translocation. Pretreatment with mdivi-1 and quercetin effectively inhibited hypoxia-induced activation of the NF-κB signaling pathway (7, 42). Hyperoside inhibits the p38 and NF-κB pathways in microglia (43). Finally, the relationship between NF-κB and HIF-1α is strongly interdependent (44). Numerous studies have demonstrated that the inhibition of inflammatory cytokines mediated by NF-κB and HIF-1α, such as AP and curcumin, significantly alleviates cerebral edema (45, 46). Conversely, NF-κB stimulates the upregulation of p62, which negatively regulates caspase-1 activation through mitophagic clearance of mitochondria that release activating signals for the NLRP3 inflammasome (47). NLRP3 inflammatory corpuscles, which contain the pyrin domain of NLR family 3 (NLRP3), play a pivotal and extensive role in orchestrating the inflammatory response (48). Neuroinflammation induced by hypoxia triggers the activation of the NLRP3 inflammasome (8). The activation of the NLRP3 inflammasome has been demonstrated to serve as a major catalyst for neuroinflammation, stimulating the secretion of proinflammatory cytokines and subsequent inflammatory responses. Research indicates that the suppression of the NLRP3 inflammasome can alleviate hypoxic–ischemic brain damage (49).

Hypoxia also promotes an inflammatory environment and triggers an inflammatory cascade in CNS tissue, which is further exacerbated by glial cells releasing proinflammatory cytokines (50). Exposure to HH leads to the activation of microglia via the p38 MAPK pathway, subsequently triggering the secretion of proinflammatory cytokines within 24 hours. This cascade ultimately results in astrocyte activation at three and seven days postexposure (36, 51). Concurrently, preexisting inflammation exacerbates hypoxia-induced injury by increasing astrocyte permeability and augmenting AQP4 activity through the Toll-like receptor 4 (TLR4), mitogen-activated protein kinase (MAPK), and NF-κB signaling pathways (52). Additionally, Song et al. demonstrated that preexisting systemic inflammation worsens hypoxic cerebral edema in a rat model via the activation of inflammatory signaling pathways through astrocyte−;microglia interactions (7).

**Figure 4:**
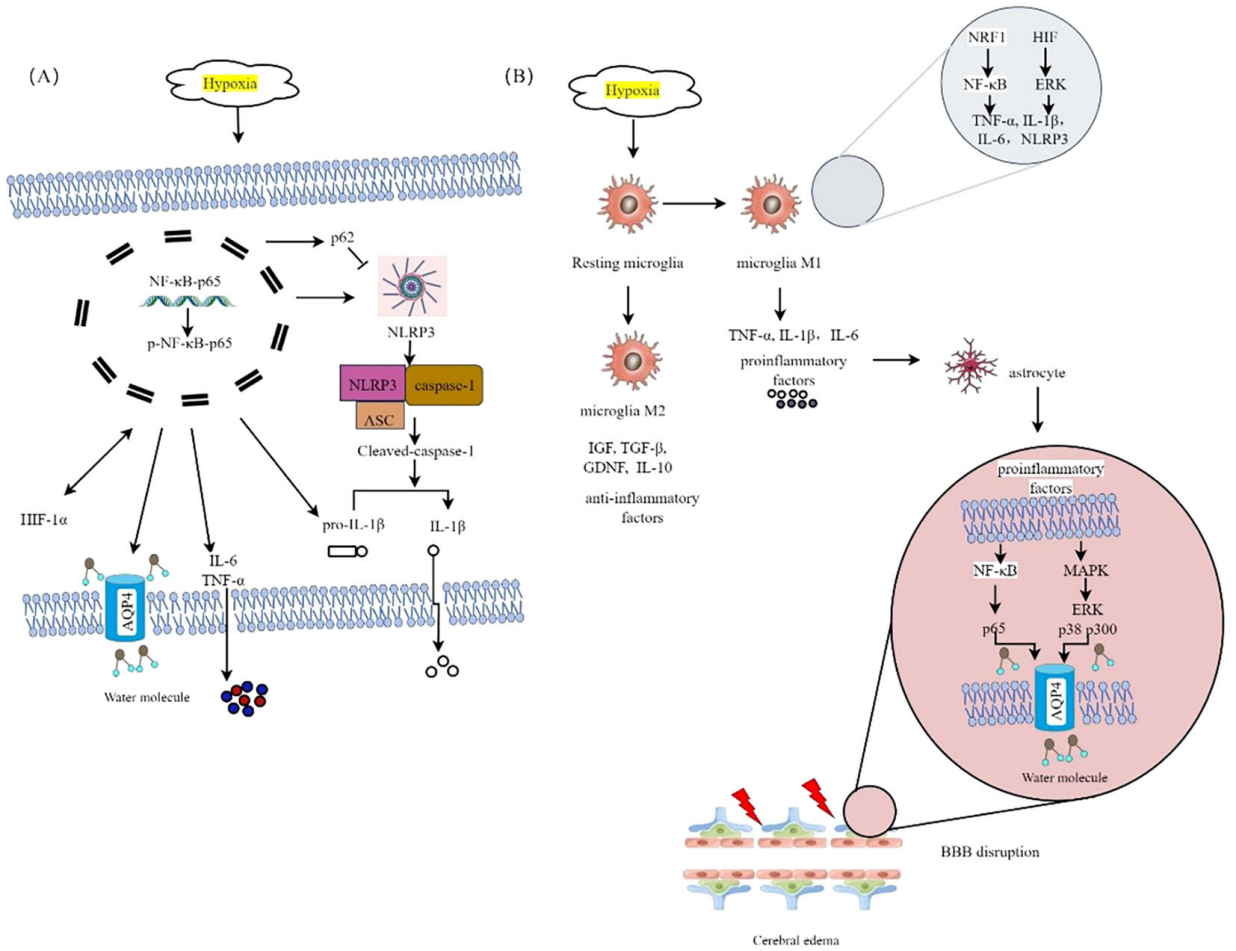
The proposed inflammatory mechanisms hypothesized to underlie HACE. **(A)** HH triggers the activation of the NF-κB signaling pathway. Hypoxia-induced NF-κB activation. Hypoxia triggers the activation of the NF-κB signaling pathway. Hypoxia-induced NF-κB activation can upregulate AQP4 expression, trigger the release of inflammatory cytokines such as IL-6 and TNF-α, activate the NLRP3 inflammasome, increase p62 expression to inhibit caspase-1 activation, and increase HIF-1α levels, which subsequently further activate NF-κB. **(B)** HACE is mediated by microglia in a hypoxic environment at high altitude. Hypoxia triggers the activation of microglia, which can exhibit distinct yet overlapping phenotypes. Specifically, the M1 phenotype is characterized by the release of proinflammatory mediators, whereas the M2 phenotype is associated with the secretion of anti-inflammatory factors. Hypoxia can promote M1 polarization of microglia by upregulating the expression of Nrf1, releasing many proinflammatory factors to activate the MAPK and NF-κB signaling pathways in astrocytes, upregulating the expression of AQP4, destroying the integrity of the BBB, and inducing HACE.

The activation of glial cells and the release of cytokines have been demonstrated to initiate and contribute to cognitive impairments in individuals with HH (36, 53). Hypoxia-induced neuroinflammation represents a potential therapeutic target for the treatment of HACE (8). Microglia, characterized by their extensively branched morphology, represent the predominant resident immune cells in the brain and serve as the primary site for NLRP3 inflammatory body expression. Upon activation, microglia undergo functional and morphological alterations (54). Exposure to HH results in a decrease in the branching complexity of microglia in the hippocampus (36). During the early stages, hypoxia induces microglial activation through the upregulation of nuclear respiratory factor 1 (NRF1), which in turn activates NF-κB p65 and TFAM, leading to an inflammatory response. Moreover, hypoxia upregulates AP2B1 and CAV-1, enhancing microglial phagocytosis of cellular debris and attenuating inflammatory bursts. However, excessive microglial activation subsequently triggers microglial migration toward blood vessels, resulting in the release of proinflammatory factors and the phagocytosis of endothelial cells, which induce endothelial damage and disrupt the integrity of the BBB, ultimately leading to astrocyte swelling. The presence of hypoxia triggers the activation of microglia, which can manifest distinct yet overlapping phenotypes characterized by proinflammatory (M1) and anti-inflammatory (M2) properties (55). M1-type microglia lead to oligodendrocyte and neuronal death by promoting the production and release of inflammatory mediators such as cytokines, chemokines, ROS, NO, and matrix metalloproteinases (56). In contrast, M2 microglia exhibit both immunosuppressive and neuroprotective properties. On the one hand, they release anti-inflammatory factors as well as neurotrophic factors (such as IGF, TGF-β, GDNF, and IL-10) to promote angiogenesis, tissue remodeling, and neurorepair (57, 58). Therefore, the regulation of microglia during brain injury is a complex process in which the beneficial or detrimental effects of microglia depend on their activation status and environmental cues (33).The polarization of microglia is a reversible process that plays a critical role in the progression, reversal, and treatment of diseases. Consequently, modulating microglial polarization to inhibit inflammation and promote tissue repair has emerged as a promising therapeutic strategy for the treatment of HACE. Wang et al. reported that stem cells derived from human exfoliated deciduous teeth (SHEDs) exhibit significant preventive and therapeutic effects on HACE by inhibiting M1-type polarization and promoting M2-type polarization of microglia via the HIF/ERK pathway (59). Studies have shown that inhibiting the NF-kB signaling pathway can effectively inhibit M1 polarization of microglia (42, 60). **Figure 4** illustrates the proposed inflammation mechanisms hypothesized to underlie HACE. Research progress on neuroinflammation in HACE patients and HACE animal models is summarized in **Table 1**.

**Table 1:** Research progress on neuroinflammation in HACE patients and HACE animal models.

	Subject	Immune cells	Immune mediators	Immune signaling pathways	References
Patient					
	3400 m, 3 nights		IL-6, interleukin-6 receptor, CRP ↑		(61)
	3833 m, 4450 m, and 5129 m		ET-1, IL-6, IL-17a ↑		(62)
	3860 m, 2 days		IL-6, IL-1β, TNF-α ↑		(7)
Animal models					
	LPS (0.5mg/kg) +6000m, 6 h, 1 d, 7 days	microglia		AQP4	(28)
	LPS (4mg/kg) +7000m, 4 h, 8 h	astrocytes/microglia	TNF-α, IL-1β ↑	TLR4/MAPKs/NF-κB, CRH/CRHR1 AQP4	(7)
	LPS (0.5mg/kg) +6000m, 6h	microglia		NF-κB microglial M1/M2 polarization	(63)
WIP1 Depletin	LPS (3mg/kg) +6000 m,10 h	microglia/macrophages	TNF-a, IL-1β, IL-6, IL-10 ↑	NF-κB	(64)
	7000 m,24 h	astrocytes	SOD ↑ TNF-α, IL-1β, VEGF, MMP-9 ↑	NFκB/VEGF/MMP-9	(37)
NLRP3 knockout mice	6000 m, 3 weeks	microglia	NLRP3, caspase-1, IL-1β,IL-6, TNF-α, iNOS ↑	NLRP3	(8)
	6000m,6h	microglia	IL-1β, IL-6, TNF-α ↑	AQP-4	(28)
	7600m, 24 h	endothelial cells		NRF1/CAV-1	(65)
	7000m, 48 h	microglia		NRF1/NF-κB p65/TFAM, CAV-1/AP2B1 phagocytic	(33)
	8000m, 3days		IL-1β, TNF-α, VEGF ↑	NF-κB/HIF-1α	(45)
	7620 m, 48 h		NF-κB, IL-1, IL-6, TNF-a, VCAM-1, ICAM-1, Pselectin, E-selectin ↑	NF-κB	(66)
	7620 m, 24 h		IL-1, IL-2, IL-18, TNF-α, P-selectin and E-selectin ↑	NF-κB	(46)
	7620m, 24 h, 48 h,7 days		IL-10 ↓ MCP-1, IL-6 and TNF- α↑		(67)
microglial cells		microglia	NO, iNOS ↑	p38 MAPK	(51)

IL-6, interleukin-6; ET-1, endothelin-1; IL-17a, interleukin-17a; IL-1β, interleukin-1 beta; TNF-α, tumor necrosis factor-alpha; AQP4, aquaporin-4; NO, nitric oxide; iNOS, inducible NO synthase; CRP, C-reactive protei; NLRP3, NLR family. ↓, decrease; ↑, increase.

## 4 Oxidative stress and HACE

The term “oxidative stress” refers to a temporary or prolonged increase in the stable concentration of ROS, which leads to the oxidative alteration of biomolecules, necrosis or apoptosis, and ultimately, cell death (68). Under normal oxygen conditions, the generation and elimination of ROS are typically maintained in a state of dynamic equilibrium. However, under HH conditions, this delicate balance is disrupted, leading to an increase in ROS levels (69). The elevation of ROS levels can lead to the accumulation of reducing equivalents in the mitochondrial electron transport chain, thereby augmenting the generation of superoxide anion radicals within the respiratory chain (70). The brain, which is highly susceptible to oxidative stress, possesses a limited capacity to endure such stress owing to its high rate of oxygen utilization, abundant iron content, substantial levels of unsaturated fatty acids, and deficiency in antioxidant defense mechanisms. Under hypoxic conditions, cerebral transvascular leakage is mediated by free radicals (5), disruption of the structural integrity of the blood−;brain barrier, cellular edema, and the initiation of an inflammatory response within the brain (6, 66, 71, 72).

### 4.1 Nrf2

**Figure 5:**
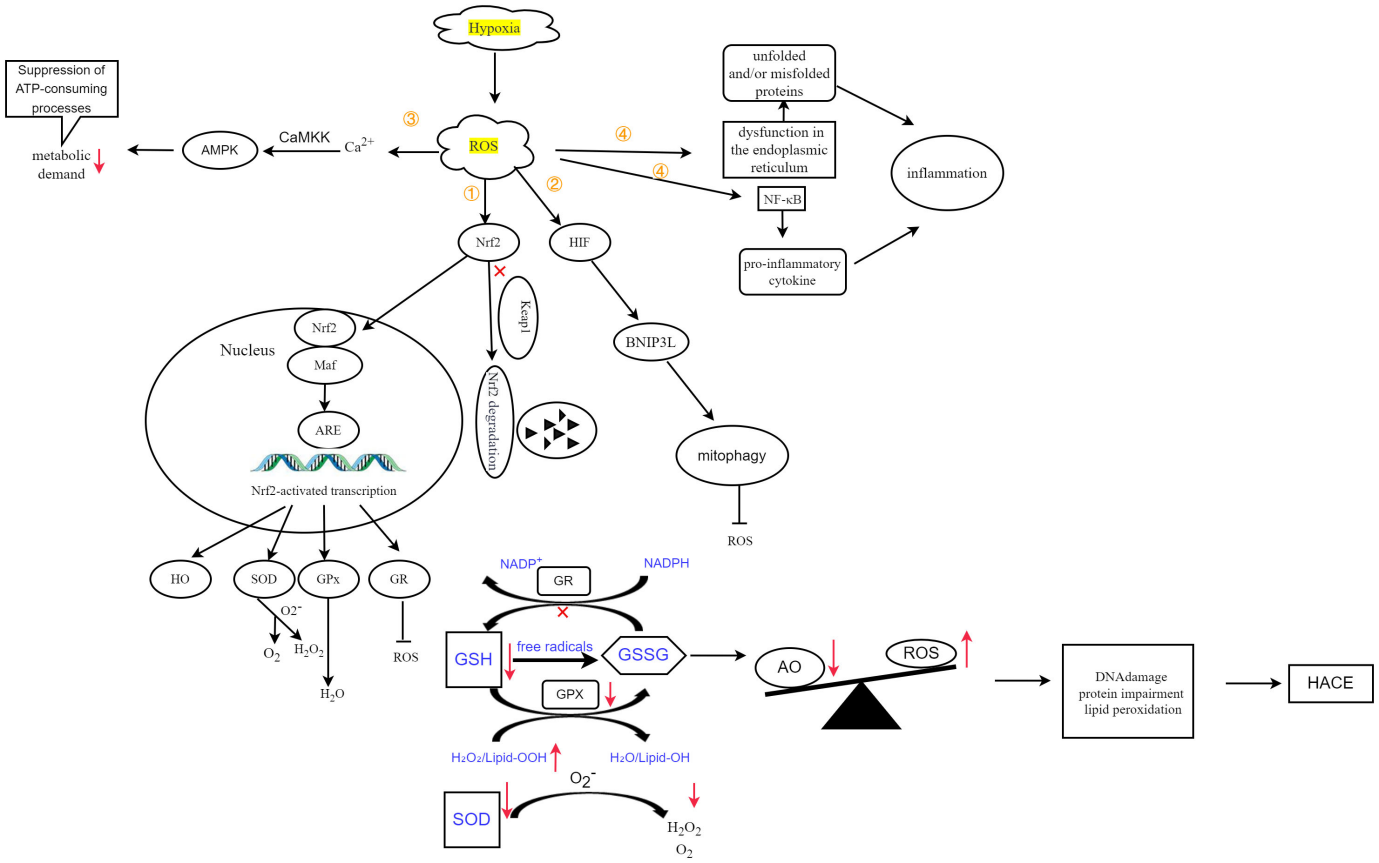
Oxidative stress, defined by an imbalance between antioxidants (AOs) and ROS, ultimately leads to DNA damage, protein dysfunction, and lipid peroxidation, thereby contributing to the development of HACE. Figure adapted from Lee et al. (25) and Burtscher et al. (3). ① Hypoxia-induced ROS inhibit the degradation of Nrf2, thereby promoting its translocation into the nucleus. Following interaction with Maf proteins, this complex subsequently activates the expression of target genes. ② Hypoxia-induced ROS activate HIF, leading to increased expression of BNIP3L and promoting mitophagy. ③ ROS can also induce Ca2+ accumulation, which in turn activates the CaMKK-AMPK pathway, thereby reducing metabolic demand. ④ ROS induce endoplasmic reticulum (ER) stress, resulting in ER dysfunction and the accumulation of unfolded or misfolded proteins, initiating inflammatory responses. Additionally, it increases NF-κB levels and stimulates the expression of proinflammatory cytokines, further triggering inflammatory responses.

The redox-sensitive transcriptional regulator nuclear factor E2-related factor-2 (Nrf2) plays a crucial role in conferring resistance against intracellular ROS by activating the transcription of antioxidant genes to modulate cellular redox homeostasis (73). When there is an adequate supply of oxygen, Nrf2 forms a complex with Kelch-like ECH-associated protein 1 (Keap1) in the cytoplasm to facilitate its degradation. However, under conditions of hypoxia-induced ROS, Nrf2 dissociates from Keap1, translocates into the nucleus, and binds to musculoaponeurotic fibrosarcoma protein (Maf). This protein subsequently interacts with antioxidant response elements and initiates the transcriptional activation of antioxidation genes, including crucial enzymes such as glutathione reductase (GR), glutathione peroxidase (GPx), superoxide dismutase (SOD) and heme oxygenase-1 (HO-1) (74). The regulatory mechanism of Nrf2 expression in response to hypoxia-induced ROS has been demonstrated (**Figure 5**).

### 4.2 The imbalance between AO and ROS

In the context of hypoxia associated with high altitude, the dysregulation of antioxidants (AOs) and ROS, as evidenced by numerous studies in both human and animal models, either due to augmented ROS production or diminished AO capacity, collectively culminates in DNA damage, protein impairment, and lipid peroxidation. To counteract the detrimental impact of ROS, superoxide dismutase (SOD) facilitates the dismutation process, transforming superoxide radicals (O_2_^-^) into oxygen molecules (O_2_) and hydrogen peroxide (H_2_O_2_), whereas glutathione peroxidase (GPx) converts H_2_O_2_ to H_2_O. Glutathione peroxidase 4 (GPX4) facilitates the reduction and detoxification of phospholipid hydroperoxides (PLOOHs) to protect against oxidative damage (75). Glutathione (GSH), an essential constituent of the cellular antioxidant system, plays a crucial role in effectively regulating the cellular levels of ROS and maintaining redox homeostasis. GSH can directly quench free radicals and ROS while also serving as a cosubstrate for GPX in the conversion of hydrogen peroxide (H2O2) and lipid peroxide (Lipid-OOH) into water and lipid alcohol (Lipid-OH), respectively. Upon reacting with oxidizing substances through either direct or enzymatic pathways, GSH undergoes oxidation to form glutathione disulfide (GSSG), which can potentially be toxic to cells. Excess GSSG can be efficiently reduced back to GSH under the catalytic action of glutathione reductase (GR) (76). The measurement of MDA, a byproduct resulting from the process of lipid peroxidation, can serve as an indicative marker for assessing lipid peroxidation levels within brain tissue. When exposed to HH, brain tissue presented increased levels of ROS and MDA, accompanied by decreased levels of the antioxidants SOD, GSH and GPx (66, 77). Antioxidant pretreatment has the potential to mitigate or even reverse oxidative stress-induced damage (77). Oxidative stress and antioxidants in HACE are summarized in **Table 2**.

**Table 2:** Oxidative stress and antioxidants in HACE.

Species	Highland conditions	Oxidation molecular markers	Antioxidant molecules	Sample	References
Human	3250 m,7 days	Protein carbonylation ↑	GSH ↓	Plasma	(78)
Rats	7620 m, 48 h	ROS, MDA ↑	GPx, SOD ↓	Brain	(66)
Rats	7600 m, 24 h	ROS, MDA ↑	GSH, SOD ↓	Brain	(79)
Rats	9144 m, 5 h	MDA ↑	GPx, GSH, SOD ↓	Brain	(77)
Rats	8000m 3 days	MDA, NO ↑	SOD, GSH ↓	Brain	(45)
Rats	25,000 ft, 24 h	ROS, MDA ↑	GSH, GPx, SOD ↓	Brain	(80)
Rats	8000m 3days	MDA, NO ↑	SOD, GSH ↓	Brain	(45)

MDA, malondialdehyde; GSH, glutathione; GPx, glutathione peroxidase; SOD, superoxide dismutase. ↓, decrease; ↑, increase.

### 4.3 Trigger inflammation

Additionally, the development of HACE pathologies is facilitated by oxidative stress-mediated activation of inflammatory pathways (5). Hypoxia induces oxidative stress, thereby causing dysfunction in the endoplasmic reticulum (81, 82), resulting in the accumulation of unfolded and/or misfolded proteins. This accumulation further disrupts inflammatory pathway regulation and accelerates various associated processes (83). Moreover, acute HH exposure in rat brains may increase oxidative stress and subsequently upregulate NF-κB levels (46, 66), inducing the expression of proinflammatory cytokines (IL-6, IL-1β, and TNF-α), as well as cell adhesion molecules (ICAM-1, VCAM-1, E-selectin, and P-selectin), thus contributing to transvascular leakage (66). The compound Mdivi-1 may mitigate the inflammatory response by modulating the ROS/NF-κB signaling pathway and suppressing AQP4 expression to alleviate cerebral edema (42).

### 4.4 Reduced metabolic demand

Additionally, under prolonged hypoxic conditions, the increase in ROS leads to an increase in calcium (Ca^2+^) levels. This subsequently triggers the activation of the CaMKK-AMPK pathway, resulting in a subsequent reduction in metabolic demand (25).

### 4.5 Autophagy enhancement

The increase in ROS results in the upregulation of HIF, which intensifies the induction of BNIP3L, leading to increased mitophagy and reduced ROS production (25).

ROS, reactive oxygen species; Nrf2, nuclear factor E2-related factor-2; Keap1, Kelch-like ECH-associated protein 1; Maf, musculoaponeurotic fibrosarcoma protein; ARE, antioxidant response elements; HO, heme oxygenase; SOD, superoxide dismutase; O2, oxygen; H2O2, hydrogen peroxide; GPx, glutathione peroxidase; GR, glutathione reductase; HIF, hypoxia inducible factor; BNIP3L, BCL2-interacting protein 3-like; CaMKK, Ca2+/calmodulin-dependent protein kinase; AMPK, AMP-activated protein kinase. AO, antioxidants; HACE, high-altitude cerebral edema; GSH, glutathione; GSSG, glutathione disulfide; NADPH, nicotinamide adenine dinucleotide phosphate; GR, glutathione reductase; Lipid-OOH, lipid peroxide; Lipid-OH, lipid alcohol; H_2_O_2_, hydrogen peroxide; SOD, superoxide dismutase; O_2_, oxygen.

## 5 Mitochondrial dysfunction and HACE

Mitochondria, the powerhouses of cells, are highly susceptible to oxygen deficiency. Hypoxia impairs mitochondrial components, mass, and dynamics, compromising cellular energy availability, altering mitochondrial function, and even leading to mitochondrial cell death (72). Consequently, mitochondria have emerged as crucial focal points in high-altitude hypoxia research (10).

**Figure 6:**
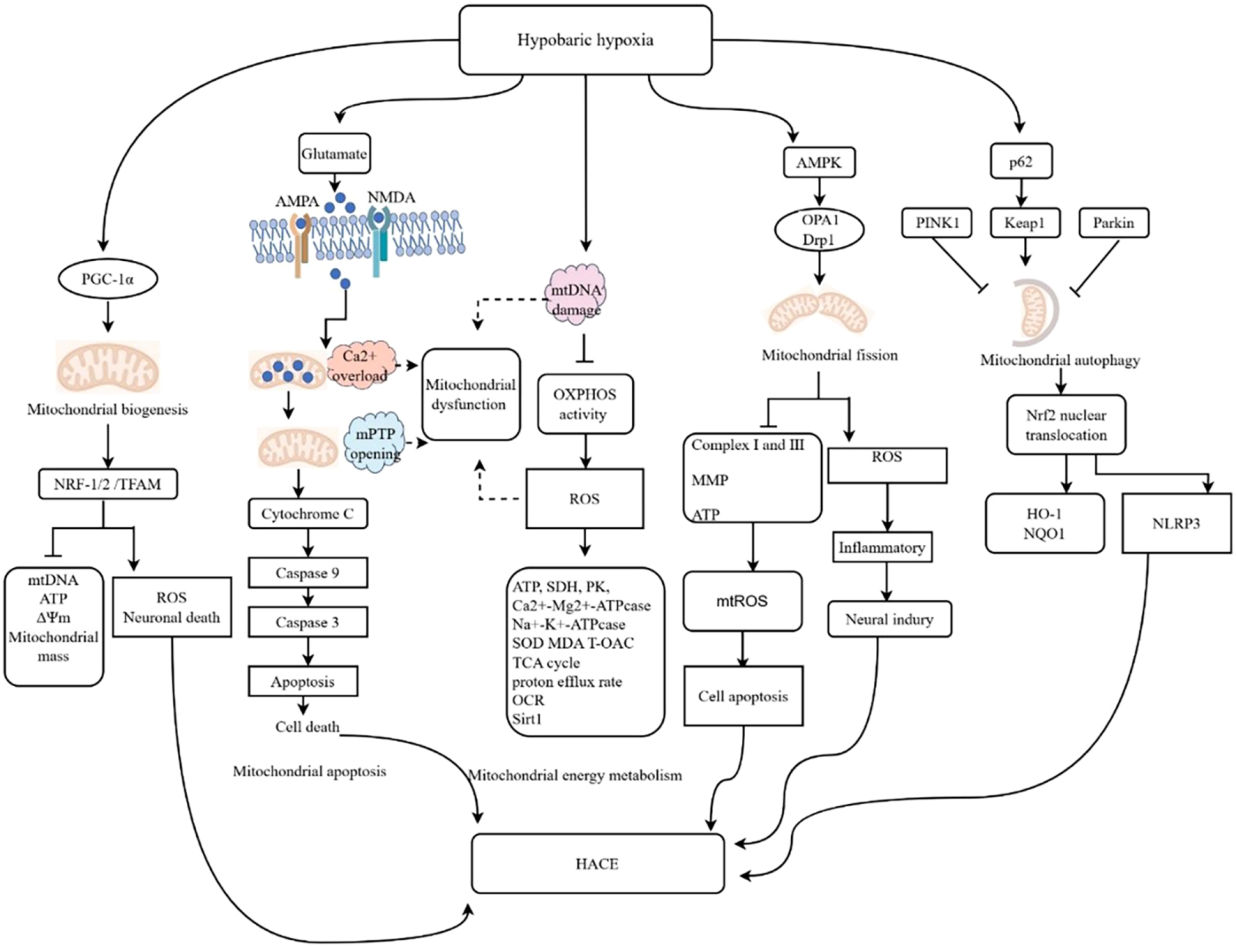
Molecular mechanisms linking mitochondrial dysfunction and HACE. The illustrated diagram delineates the five primary pathways through which mitochondrial dysfunction influences the cellular environment in the context of HH. These pathways include energy metabolism, mitochondrial biogenesis, mitochondrial apoptosis, mitochondrial dynamics, and mitophagy. Additionally, the diagram elucidates the specific molecular mechanisms underlying each pathway. The activation of AMPA and NMDA receptors leads to mitochondrial calcium (Ca2+) overload, resulting in the opening of the mitochondrial permeability transition pore (mPTP), which subsequently activates the caspase cascade and induces cell death. Damage to mitochondrial DNA (mtDNA) results in decreased oxidative phosphorylation (OXPHOS) activity and increased ROS levels. Calcium overload, mPTP opening, mtDNA damage, and oxidative stress are potential contributors to mitochondrial dysfunction following HACE. ↑, promotional effect; ⊥, inhibitive effect. OCR, oxygen consumption rate MCU, mitochondrial calcium uniporter; MPTP, mitochondrial permeability transition pore.

Mitochondrial dysfunction is a severe adverse consequence of abnormal oxygen metabolism. During hypoxia associated with high altitudes, the mitochondrial electron transport chain (ECT) generates an excessive amount of ROS, leading to detrimental effects on cellular function and integrity. The overproduction of ROS can induce mutations in mitochondrial DNA, initiate lipid peroxidation, and trigger the opening of mitochondrial membrane channels. Consequently, these events result in the collapse of the mitochondrial membrane potential, further exacerbating ROS accumulation and disrupting mitochondrial permeability. As a consequence, impaired mitochondrial function ensues. **Figure 6** illustrates the molecular mechanisms linking mitochondrial dysfunction and HACE.

### 5.1 Mitochondrial energy metabolism

Increasing evidence has provided substantial support for the critical involvement of mitochondrial energy metabolism in HH-induced brain injury. HH leads to the downregulation of specific mitochondrial proteins that play a regulatory role in brain energy production, such as the F1-ATPase beta-subunit and electron transfer flavoprotein alpha-subunit (84). Concurrently, disruption of mitochondrial energy metabolism, including failure of membrane ion pumps and efflux of neuronal K+ (38, 85), occurs.

### 5.2 Mitochondrial biogenesis

Peroxisome proliferator-activated receptor gamma coactivator 1 alpha (PGC-1α), a pivotal regulator that governs mitochondrial biogenesis, initiates the transcription and replication of mtDNA by activating nuclear respiratory factor (NRF-1/2) and mitochondrial transcription factor A (TFAM). Consequently, this induces the interaction between TFAM and crucial mitochondrial enzymes to fulfill energy requirements in response to environmental changes (86, 87).

### 5.3 Mitochondrial apoptosis

Under physiological conditions, the mitochondrial permeability transition pore (mPTP) plays a crucial role in facilitating ATP synthesis through oxidative phosphorylation, thereby maintaining the mitochondrial membrane potential and ensuring both the intracellular and the extracellular ion balance. In the context of brain injury, excessive release of glutamate activates NMDA and AMPA receptors, which leads to a significant increase in the cytoplasmic Ca2+ concentration. To counteract this, mitochondria absorb excess Ca2+, aiming to preserve cytoplasmic Ca2+ homeostasis. However, this absorption results in mitochondrial Ca2+ overload, which subsequently triggers opening of the mPTP. The prolonged opening of the mPTP impairs the mitochondrial respiratory chain and increases the production of ROS. Over time, this condition causes morphological changes in mitochondria, such as swelling, and compromises their functionality. Ultimately, it initiates the endogenous apoptotic pathway by promoting the release of cytochrome C, thereby activating the caspase cascade (87). Sal treatment can mitigate HACE by reducing Ca2+ levels, preventing opening of the mPTP, and inhibiting cellular apoptosis (88). Bax, a proapoptotic protein, increases mitochondrial membrane permeability and facilitates the release of caspase-9 and caspase-3. Conversely, Bcl-2, an antiapoptotic protein, reduces mitochondrial membrane permeability and inhibits mitochondrial apoptosis. The ratio of Bax/Bcl-2 is considered a crucial marker for pro- or antiapoptotic activity, and it increases following exposure to HH (10, 85). Therefore, targeting mitochondrial apoptosis could be a potential therapeutic strategy for high-altitude cerebral hypoxia (10).

### 5.4 Mitochondrial dynamics

The process of mitochondrial dynamics, which is characterized by coordinated cycles of fission and fusion, significantly impacts mitochondrial morphology, biogenesis, and subcellular localization and distribution, as well as cellular bioenergetics, injury, and apoptosis. In high-altitude cerebral hypoxia, electron microscopy revealed the presence of abnormal mitochondria with distinct swelling, unclear or disrupted membranes, and reduced cristae (89–91). Furthermore, when exposed to high-altitude hypoxia, mouse hippocampal neurons exhibit decreased mitochondrial length, increased fragmentation of mitochondria, and decreased membrane potential and oxidative phosphorylation, resulting in increased mitochondrial fission and dysfunction (10). High-altitude exposure induces increased phosphorylation of Dynamin-related protein-1 (Drp1), a key regulator of mitochondrial division, resulting in excessive mitochondrial fission and subsequent fragmentation. Treatment with mdivi-1 effectively inhibits Drp1 phosphorylation, thereby preventing mitochondrial fragmentation and glial cell activation and ultimately alleviating cerebral edema in mice (42). The inhibition of the mitochondrial fusion/fission-related proteins Drp1 and Fis1 plays a crucial role in significantly mitigating cerebral edema after HACE (86). The proteins Drp1 and OPA1 play crucial roles in maintaining mitochondrial dynamics homeostasis and preventing mitochondrial damage in HACE (88).

### 5.5 Mitochondrial mitophagy

Mitochondrial autophagy, or mitophagy, is a specific mechanism that selectively eliminates damaged mitochondria. This process is crucial for maintaining mitochondrial homeostasis and cellular survival, particularly in postmitotic neurons. PINK1 and Parkin are the two most representative proteins involved in mitophagy (25).

## 6 Oxygen metabolism and HACE treatment

The prevention of HACE is of paramount importance for military operations and economic progress. A recommended approach to prevent HACE involves a gradual ascent with sufficient time allocated for acclimatization. However, prophylactic medication remains the predominant method. Research has revealed that acute HH-induced oxidative stress can contribute to the development of HACE. The primary therapeutic modalities against oxidative stress include preventing oxidant production, inhibiting redox signaling that leads to inflammation or cell death, and increasing the levels of antioxidant enzymes and their substrates. Antioxidants play crucial roles in combating oxidative stress and are being explored as promising therapeutic strategies to alleviate hypoxia-induced HACE (5). It has been documented that various antioxidants such as flavones naringenin and quercetin (80, 86, 92, 93) 5,6,7,8-trtrahydroxyflavon (5,6,7,8-THF), synthetic derivatives of flavones (94), withania somnifera root extract (95), sea buckthorn seed oil (77), ginkgolide B (96), acetyl-L-carnitine (97, 98), polysaccharide derived from Potentilla anserina L (PAP) (45), alga Spirogyra porticalis (99), and dihydromyricetin (100) have the potential to alleviate cerebral dysfunction induced by HH.

Nrf2 antioxidants can activate the Nrf2 pathway, which plays a crucial role in regulating oxidative stress and neuroinflammation. Substantial advancements have been made in the field of research on Nrf2 activators and drugs associated with antioxidant mechanisms, as they are considered promising pharmaceutical agents for enhancing antioxidant capacity and mitigating the progression of HACE. Gong et al. demonstrated that ganglioside GM1, a neuroprotective sphingolipid, alleviates the severity of HACE by attenuating oxidative stress and the inflammatory response through activation of the PI3K/AKT-Nrf2 pathway in HACE patients (79). Additionally, acetyl-L-carnitine (ALCAR) mitigates Nrf2 degradation and enhances Nrf2 nuclear translocation, thereby facilitating the transcription of Nrf2-regulated antioxidant genes through the TrkA/ERK1/2/Nrf2 pathway, which confers neuroprotective effects against oxidative stress on hippocampal neurons (98). A preliminary, underpowered study suggested potential benefits of antioxidants in preventing acute mountain sickness (101); however, these findings were not supported by a subsequent larger randomized controlled trial (102). The limited body of research currently available restricts the ability to draw definitive conclusions (5, 103).

The involvement of inflammation in the pathogenesis of HACE suggests that targeting inflammatory processes could be a viable strategy for mitigating its development. Animals preconditioned with sphingosine-1phosphate (S1P), a bioactive lipid, demonstrated protection against inflammation, vascular permeability, oxidative damage, and brain tissue injury during acute and subchronic HH (67). Furthermore, the NF-κB signaling pathway plays a crucial regulatory role in the pathogenesis of HACE, making it a promising therapeutic target. Pretreatment with curcumin significantly attenuated hypoxia-induced cerebral transvascular leakage, accompanied by a concurrent decrease in brain NF-κB levels (66). Subsequently, Sarada et al. proposed that curcumin prophylaxis preserves the integrity of tight junction proteins and enhances ion channel expression in the brains of rats exposed to hypoxia through the modulation of oxygen-dependent NF-κB and HIF-1α transcripts, which regulate adaptive responses such as Na+/K+-ATPase and ENaC (46). Furthermore, THC, a major bioactive metabolite of curcumin, may exert a prophylactic effect through inhibition of the NF-κB/VEGF/MMP-9 pathway (37). Studies have documented the potential of Puerarin (40), PhGCs (104), polysaccharides derived from Potentilla anserina L. (PAP) (45), GP-14, a newly identified dammarane-type saponin (63), salidroside (105), Rhodiola crenulate extract (38), epicatechin gallat (60) and exendin-4 (106) for preventing and/or treating HACE by inhibiting the NF-κB signaling pathway. The inhibition of microglial NF-κB signaling effectively suppresses M1-type polarization, offering novel insights into the prevention of HACE through the modulation of microglial polarization (60, 63).

The maintenance of mitochondrial functional stability and activation of the HIF-1 signaling pathway are attractive and feasible targets for the prevention or alleviation of HACE pathology (9, 107). Rhodiola crenulata can regulate HIF-1α-mediated processes related to mitochondrial energy to attenuate neuronal apoptosis, thereby protecting rats from brain damage at high altitudes (85). The dynamin-related protein-1 (Drp1) inhibitor Mdivi-1 directly reduces mitochondrial fission, decreases AQP4 expression, decreases IL-6 and TNF-α secretion, and alleviates cerebral edema in mice. It shows promise as a potential molecule for treating HACE (42). Quercetin inhibits microglial activation to alleviate neurotoxicity through the interplay between the NLRP3 inflammasome and mitophagy (108). Dihydromyricetin is a flavonoid derived from natural sources that attenuates oxidative stress while promoting mitochondrial biogenesis and enhancing synaptic function through activation of the SIRT3/FOXO3 signaling pathway (100). The administration of Rhodiola crenulata and its active ingredient Sal preserves blood−;brain barrier integrity and enhances energy metabolism by activating the AMPK/Sirt1 pathway, inhibiting Drp1 activation, and promoting OPA1 expression (88). The HIF-1α/microRNA 210/ISCU1/2 (COX10) signaling pathway is involved in the regulation of apoptosis and mitochondrial energy metabolism by Rhodiola crenulata (85). Treatment strategies for HACE patients and HACE animal models are summarized in **Table 3**.

**Table 3:** Treatment HACE patients and HACE animal models.

Drugs	Compound property	Subject	Immune mediators	Mechanism of action	References
Naringenin and quercetin (10 mg/kg)	flavones	7800m, 24 h	HIF1a,VEGF, active caspase 3 and ubiquitin levels ↓	downregulation of caspase-3 and ubiquitinated proteins	(92)
Quercetin (50,75,100mg/kg) 7 days	flavonoid	5000m,7 days	Sirt1, PGC-1α,FNDC5, BDNF ↑	PGC-1α/FNDC5/BNDF Sirt1/PGC-1α/Nrf-1/Tfam antioxidative,mitochondrial biogenesis and dynamics, metabolic regulator	(86)
Quercetin (50 mg/kg 1 day pre-exposure and 7 days)	flavonoid	7600m, 7days	ROS, MDA, GSSG↓ GSH,GR,Superoxide dismutase ↑ GPx↓ caspase-3 ↓	antioxidative and anti-apoptotic	(93)
Quercetin (50 mg/kg body) for 1 day	flavonoid	25,000 ft, 24 h	ROS, MDA ↓ GPx, GSH, SOD↑ NF-kB ↓	antioxidative and anti-inflammatory	(80)
5,6,7,8-THF (500mg/kg/d) for 5 days	flavonoids flavones	8000m, 12h	SOD,CAT,GSH-Px↑ ATPase activity(Na+-K+-ATPase and Ca2+-Mg2+-ATPase) ↑ H2O2, MDA, LDH ↓	antioxidative and energy metabolism	(94)
Withania somnifera root extract (200 mg/kg BW) 21 days and 07 days	antioxidant	25,000 ft, 7days	ROS, MDA, NOS ↓ GSH, SOD ↑ Bax ↓ BCl2 ↑ BDNF and NCAM ↑	NO-cyclooxygenase-prostaglandin signaling NO, corticosterone, oxidative stress and AchE activity	(95)
seabuckthorn seed oil(2.5ml/kg b.w.) for 12h	valuable plant natural oil	9144m, 5 h	MDA↓ GSH,GR,GPx, SOD ↑	preventing transvascular fluid leakage through stabilization of the antioxidant defense system	(77)
Ginkgolide B (6 and 12mg/kg) for 3 days	ginkgolides and terpenoid	8000 m, 24 h	SOD, GSH ↑ MDA,caspase-3, PARP ↓	the antioxidant properties and inhibition of caspase-dependent neuronal damage	(96)
acetyl-L-carnitine (75 mg/kg for 3 days)	NMDA receptor.	6100m, 3days	GSH ↑ GSSG,MDA,LDH ↓	attenuated oxidative stress and suppressed the apoptotic cascade	(97)
ALCAR (75 mg/kg for 14 days)	NMDA receptor.	25,000 ft (7620 m),14days	ROS, MDA ↓ SOD, GR, GSH ↑ GPx, GSSG ↓ Thioredoxin,TrkA ↑	TrkA/ERK1/2/Nrf2 TrkA-mediated activation of ERK1/2 leads to an enhancement in the transcription of antioxidant genes regulated by Nrf2	(98)
PAP (100, 200, and 400 mg/kg) for 3 days	polysaccharides	8000m, 3days	MD, NO ↓ SOD, GSH ↑ IL-1β,TNF-α,VEGF ↓ NF-κB, HIF-1α ↓	NF-κB/HIF-1α antioxidative and anti-inflammatory	(45)
algal extract (600 µg/ml)	filamentous alga	–	Catalase, SOD GSH ↑	antioxidative	(99)
Dihydromyricetin(100 mg/kg for 7days)	flavonoid	5000m, 7days	ROS, MDA ↓ ATP ↑	mitochondrial biogenesis and synaptic function via SIRT3/FOXO3 signaling	(100)
Ganglioside GM1 for 3 days	sphingolipid	7600 m, 24 h	brain vascular leakage, aquaporin-4, Na+/K+-ATPase ↓ occludin ↑ ROS, MDA ↓ SOD, GSH ↑ IL-1β, TNF-α, and IL-6 ↓	PI3K/AKT-Nrf2 antioxidative and anti-inflammatory	(79)
S1P (1µg/kg) for 3 days	blood borne lipid	7620m, 24 h, 48 h and 7 days	IL-10 ↓ MCP-1, IL-6 and TNF- α ↑ ATP ↑	protected against vascular leakage in critical organs, oxidative damage, and inflammatory responses	(67)
curcumin (100mg/kg) 1 h	iferulomethane	7620 m, 24 h	ROS, MDA ↓ GPx, SOD↑ NFkB ↓	antioxidant and anti-inflammatory	(66)
curcumin (100 mg/kg body weight) 1 h	diferulomethane	7620 m, 24 h	NF-κB (IL-1, IL-2, IL-18 and TNF-α↓), P-selectin and E-selectin ↓ IL-10 ↑, Hif-1α,VEGF ↓ Na+/K+-ATPase, ENa↑ ZO-1, JAMC, claudin 4 and claudin 5↑	antioxidative and anti-inflammatory	(46)
Tetrahydrocurcumin (THC) (40 mg/kg) for 3 days	bioactive metabolite of curcum	7000 m, 24 h	SOD ↑ TNF-α,IL-1β,VEGF,MMP-9 ↓	NF-κB/VEGF/MMP-9	(37)
Puerarin 100 mg/kg for 6 days	isoflavones	5000 m, 48 h	AQP1, AQP4, NF-κB ↓ TNF-α a, IL-1β↓	NF-κB/AQP inhibition of the NF-κB signaling pathway	(40)
PhGCs (50, 100, and 200 mg/kg) for 3 days	Phenylethanoid glycosides	8000m, 3days	SOD, GSH ↑ MDA ↓ NF-κB, IL-1β, TNF-α ↓	antioxidative and inhibition of the NF-κB signaling pathway	(104)
GP-14 (100 and 200 mg/kg) for 7 days	Gypenosides a newly identified dammarane-type saponin	LPS(0.5 mg/kg)+6000m, 6h	IL-6, IL-1β ↓ CD16/32 ↓ CD206 ↑ ZO-1↑ endothelial cell markers CD31 and IgG ↓	M1/M2 microglial polarization transformation inhibiting the NF-κB signaling pathway	(63)
Salidroside (20 and 50 mg/kg) for 7 days		3,000 m,30min 4,500 m,30min 9,000 m, 24h	ROS MDA ↓ SOD, GSH-Px ↑ Na+-K+-ATPase, Ca2+-Mg2+ATPase, ATP, SDH, HK,PK ↑ LDH, LD ↓ ZO-1, Occludin, Claudin-5 ↑ TNF-α, IL-1β and IL-6 ↓ p–NF–κB-p65, NLRP3, cleaved-Caspase-1, ASC ↓ iNOS and COX2 ↑	inhibiting NF-κB/NLRP3 pathway	(105)
RCE (0.5, 1.0 and 2.0 g/kg) for 7 days	Rhodiola crenulate extract	8000m, 48 h.	SOD, GSH-Px T-AOC ↑ MDA, LDH ↓ ATP, SDH, PK, Ca2+-Mg2+-ATPcase and Na+-K+-ATPcase ↑ LA ↓ tight junction proteins (ZO-1, claudin-5 and occludin) ↑ IL-6, IL-1β and TNF-α ↓ p-p65/p65, ASC, NLRP3, cleaved-caspase-1/caspase-1, IL-18 ↓	NF-κB/NLRP3 antioxidative, energy metabolism and anti-inflammatory	(38)
epicatechin gallate (ECG) 100 mg/kg 1h pre-exposure	Catechins, polyphenols	7000 m,48h	p-p65, NLRP3, TNF-α, IL-1β↓ AQP4↓	ameliorated neuroinflammation and inhibited the activation of NF-κB signaling pathway as well as microglial proliferation and activation	(60)
Exendin-4 (2, 10 and, 100 μg) 3days	peptide	7000m, 3days	GSH, SOD ↑ MDA ↓ Occludin and ZO-1↑ AQP4 ↓ IL-6,TNF-α, NF-κB ↓ SOCS-3, EPAC1 ↑	inhibit oxidative stress and inflammatory responses, safeguard the integrity of BBB through cAMP/EPAC1/SOCS-3 signaling pathway	(106)
RCAE (0.315, 0.63, 1.26 g/kg/day) for 7 days	Rhodiola crenulata	3,000m,30min 4,500m,30min 9,000 m, 24h	Apaf-1, Bax, Cyto-c, Caspase-3, MDA, LDH,GSSG ↓ GSH, SOD,HIF-1α, ISCU1/2, COX10, and Bcl-2 ↑	HIF-1α/microRNA 210/ISCU1/2(COX10) apoptotic and mitochondrial energy metabolism	(85)
mdivi-1 (20 mg/kg) for 3 days	quinazolinone derivative	5000 m,48h 7600 m, 24h	TNF-α,IL-6 ↓ ROS ↓AQP4 ↓	ROS/NF-κB mitochondrial fragmentation, activation of glial cells, and anti-inflammatory responses	(42)
R. crenulate extract (RCE, 0.5, 1.0 and 2.0 g/kg) and salidroside (Sal, 25, 50 and 100 mg/kg) for 7 days	Rhodiola crenulata	3,000 m,30min 4,500 m,30min 8,000 m, 24h	Claudin-1, ZO-1, occludin ↑ SOD, GSH-PX, SDH ↑ LDH,MDA ↓ ROS, Ca2+ ↓ OPA1 ↑ MCU, p-Drp1ser616, Sirt 1 ↓ p-AMPKα, p-AMPKβ ↑	maintaining BBB integrity and enhancing energy metabolism through activation of the AMPK/Sirt1 signaling pathway	(88)
Eleutheroside B (100 mg/kg or 50 mg/kg) for 3 days	a key component of Eleutherococcus senticosus,	6000m, 10 days	ROS and MDA ↓ GSH ↑ IL1β, IL-6, and TNF-α↑	antioxidant stress and anti-neuroinflammatory effects by inhibiting the JAK2/STAT3 signaling pathway	(109)

HIF1a, hypoxia inducible factor 1a; VEGF, vascular endothelial growth factor; Sirt1, sirtuin 1; FNDC5, fibronectin type III domain-containing protein 5; BDNF,brain-derived neurotrophic factor; ROS, reactive oxygen species; MDA, malondialdehyde; SDH, succinate dehydrogenase; GSSG, glutathione disulfide; GSH, glutathione GR, glutathione reductase; GPx, glutathione peroxidase;SOD, superoxide dismutase; 5,6,7,8-THF, 5,6,7,8-trtrahydroxyflavone; H2O2, hydrogen peroxide; CAT, catalase; GSH-Px, glutathione peroxidase; LDH,lactate dehydrogenase; NO, nitric oxide; TNF-α, tumor necrosis factor-alpha; PARP, poly(ADP-ribose) polymerase; ALCAR, acetyl-L-carnitine;PAP, Potentilla anserina L polysaccharide; TrkA, tyrosine kinase A; ERK1/2,extracellular related kinase; Nrf2, Nuclear factor erythroid 2-related factor 2; IL-6, interleukin-6; FOXO3, Forkhead box-O3;S1P, sphingosine-1-phosphate; ENaC, endothelial sodium channel; JAMC, junctional adhesion molecule C; IL-1β, interleukin-1 beta; AQP4, aquaporin-4;PhGCs, Phenylethanoid glycosides; GP-14, gypenoside-14; NLRP3, NOD-like receptor protein 3; RCE, Rhodiola crenulate extract; LA, lactic acid; ATP, adenosine triphosphate; iNOS, inducible NO synthase; ECG, epicatechin gallate; RCAE,Rhodiola crenulata aqueous extract; BBB, blood-brain barrier; ISCU1/2, iron-sulfur cluster scaffold; COX10, cytochrome c oxidase assembly protein; mdivi-1, mitochondrial division inhibitor-1. ↓, decrease; ↑, increase.

## 7 Conclusion

In this study, we systematically elucidated the mechanisms of oxygen metabolism in HACE, including hypoxia, oxidative stress, and mitochondrial dysfunction. This comprehensive analysis may enhance our understanding of the disease. Abnormal oxygen metabolism plays a pivotal role in influencing the occurrence and progression of HACE. Therefore, in-depth research into key molecular mechanisms associated with oxygen metabolism could uncover new diagnostic and therapeutic targets for HACE. An integrated approach may be necessary to address the multiple pathogenic mechanisms of HACE. Disordered oxygen metabolism serves as a bridge connecting various pathogenic factors of HACE and is an integral part of these mechanisms. We examined alterations in oxygen metabolism during the pathogenesis of HACE, in which the activation and expression levels of ROS, HIF-1α, Nrf2, NF-κB, NLRP3 and AQP4 are critical factors. Several potential therapeutic agents targeting these pathways have been investigated. These treatment strategies demonstrate feasibility and provide novel insights into the prevention of HACE, showcasing promising clinical application prospects. However, further validation through larger randomized controlled trials and a more extensive body of research is necessary. Numerous factors affect oxygen metabolism, including environmental and individual factors. Further exploration of oxygen metabolism-related treatments is essential to correct metabolic abnormalities in HACE. The central role of oxygen metabolism in HACE provides a novel avenue for discovering new anti-HACE treatments.
